# Targeted Temperature Management for Out-of-Hospital Cardiac Arrest Survivors

**DOI:** 10.7759/cureus.69204

**Published:** 2024-09-11

**Authors:** Noor ul Huda Ramzan, Rubaid A Dhillon, Mian Uman Anwer, Muhammad Bilal Hashmat, Khadija Shahjahan, Talha Asif, Ahmed Sadain Khalid, Fahad Saleem

**Affiliations:** 1 Medicine, University Medical and Dental College, Faisalabad, PAK; 2 Medicine, Riphah International University, Rawalpindi, PAK; 3 Medicine, Allied Hospital, Faisalabad, PAK; 4 Medicine, Faisalabad Medical University, Faisalabad, PAK; 5 Medicine, Punjab Medical College, Faisalabad, PAK; 6 Medicine, Shifa Medical Center, Jhang, PAK

**Keywords:** out-of-hospital cardiac arrest, neurological outcomes, randomized clinical trial, targeted temperature therapy, induced hypothermia

## Abstract

Targeted temperature management (TTM), specifically therapeutic hypothermia, has been proposed to provide neuroprotective and mortality benefits for out-of-hospital cardiac arrest (OHCA) survivors. This proposition was based on small-scale trials from the early 2000s, leading to its incorporation into various international guidelines. The proposed neuroprotective mechanisms include reducing cerebral metabolic rate, stabilizing the blood-brain barrier, reducing the release of excitatory neurotransmitters, and suppressing apoptotic pathways. However, these early trials have been criticized for their high risk of bias and lack of standardized protocols. Recent evidence from more rigorously controlled randomized trials indicates no significant association between hypothermia and improved neurological outcomes or survival rates. This review explores the latest clinical evidence on TTM for OHCA patients, discussing the pathophysiology, evaluating the effectiveness of hypothermia through various clinical trials, and providing recommendations for future research and clinical practice.

## Introduction and background

Cardiac arrest is the sudden and unexpected loss of the heart's pumping function, halting blood flow to critical organs such as the brain and heart. Over 650,000 adults in the United States experience cardiac arrest each year [1]. The survival rate for patients who experience out-of-hospital cardiac arrest (OHCA) is close to 10% [2]. In contrast, the survival rate for in-hospital cardiac arrest (IHCA) is estimated to be 25% [3]. Consequently, sudden cardiac death is a significant cause of mortality in the developed world. Even among survivors, there is a considerable risk of neurological disability and poor quality of life, with severe fatigue found in 52%, post-traumatic stress disorder in 28%, anxiety and depression in 15%, and cognitive deficits in 13% of survivors [3].

Current cardiac arrest management revolves around early resuscitation to restore blood flow to the heart and brain and prevent further damage to these vital organs. Management can be divided into two broad categories: basic life support (BLS) and advanced cardiac life support (ACLS) [4]. BLS involves immediately recognizing cardiac arrest, calling for emergency medical services, and prompting bystander cardiopulmonary resuscitation [5]. In contrast, ACLS involves using advanced interventions such as defibrillation, advanced airway management, and medications to restore heart rhythm and improve blood flow to the body [5]. This may also involve identifying and treating any underlying causes of cardiac arrest, such as electrolyte imbalances or cardiac ischemia.

Although successful early resuscitation is crucial in managing cardiac arrest, survival does not necessarily equate to full recovery. It carries a high risk of poor long-term outcomes and high mortality rates. As such, post-resuscitative care to improve neurological and functional outcomes and reduce the risk of subsequent cardiac events is essential in managing cardiac arrest survivors. One component of post-resuscitative care involves inducing hypothermia to provide neuroprotection and reduce mortality.

Therapeutic hypothermia was proposed to offer neuroprotective and mortality benefits for OHCA survivors based on two small-scale trials from the early 2000s [6]. Consequently, therapeutic hypothermia was incorporated into various guidelines from international bodies. The 2021 European Resuscitation Council (ERC), European Society of Intensive Care Medicine (ESICM), and the International Liaison Committee on Resuscitation (ILCOR) advanced life support task force recommend maintaining a constant targeted temperature management (TTM) of 32°C to 36°C as soon as possible after the return of spontaneous circulation following cardiac arrest [6-8]. However, key international cardiology societies, including the ERC, ESICM, and the ILCOR, have raised concerns about the high risk of bias and nonstandardized protocols in these early trials, prompting a reevaluation of the evidence supporting therapeutic hypothermia [7].

Recent evidence from more rigorously controlled randomized controlled trials has shown that there is no significant association between hypothermia and favorable neurological outcomes or increased survival rates. Therefore, healthcare providers face significant challenges in providing appropriate post-resuscitative care to cardiac arrest survivors, with variable practices and outcomes across different settings [9]. There is a pressing need for more research into post-resuscitative care for cardiac arrest patients to develop robust evidence-based guidelines and improve patient outcomes. As such, this review will focus on one aspect of postcardiac arrest care: TTM in OHCA survivors. The review aims to discuss the pathophysiology of how targeted hypothermia may produce beneficial outcomes, take an in-depth look at various clinical trials evaluating the effectiveness of this treatment modality, and explore the future of TTM, providing clinicians with recommendations.

## Review

Physiology of neuroprotection due to hypothermia

Although the exact mechanism is still unclear, there are many pathways by which hypothermia is hypothesized to produce beneficial effects. The proposed mechanisms are explained in Table 1.

**Table 1:** Mechanisms of neuroprotection by hypothermia IL: interleukin; TNF: tumor necrosis factor

Mechanism	Detail
Reduction in cerebral metabolic rate and oxygen consumption	By lowering the body's core temperature, hypothermia can reduce the brain's metabolic rate and oxygen consumption [10]. This reduction helps decrease the demand for oxygen and other nutrients, which can preserve brain tissue and minimize damage following a cardiac arrest
Preservation of the blood-brain barrier	During a cardiac arrest, the blood-brain barrier can be compromised, allowing inflammatory cytokines and free radicals to cross the barrier freely and cause damage [11]. Hypothermia stabilizes the blood-brain barrier by reducing inflammation and oxidative stress, which are primary contributors to blood-brain barrier disruption [11]
Reduced production of free radicals and inflammatory cytokines	Hypothermia inhibits the production of free radicals by inhibiting superoxide and lipid peroxide [12]. Additionally, acute neuronal injury during cardiac arrest increases inflammatory cytokines IL-1β, IL-6, IL-18, and TNF, exacerbating neuronal damage [13]. Hypothermia reduces the production of these inflammatory cytokines while simultaneously increasing the production of anti-inflammatory cytokines [14]
Reduction in the release of excitatory neurotransmitters	Following a cardiac arrest, there can be an increase in the release of excitatory neurotransmitters, such as c-fos and glutamate, which can cause further damage to neurons [15]. Hypothermia reduces the release of excitatory neurotransmitters, helping to prevent further neuronal damage and promote healing [16]
Suppression of apoptotic pathways	Hypothermia suppresses both the intrinsic and extrinsic apoptotic pathways, which can help prevent cell death in the brain and other vital organs [17,18]

Methods

A detailed literature search was performed across PubMed, Embase, Web of Science, and the Cochrane Library databases to identify studies published between January 1, 2000, and July 31, 2024. The search focused on English-language, human-based studies. The following search terms and Medical Subject Headings were employed in various combinations: TTM, therapeutic hypothermia, OHCA, and TTM trials, along with mortality, neurological outcomes, and postcardiac arrest care.

Studies meeting inclusion criteria were randomized controlled trials, observational studies, and systematic reviews that evaluated the effectiveness of TTM in OHCA survivors, with a specific focus on mortality and neurological outcomes. Exclusion criteria comprised studies conducted in languages other than English, those with an animal focus, those primarily addressing IHCA, or those lacking data on the key outcomes of interest. All articles were screened by title and abstract for relevance, followed by a full-text review of those that met the inclusion criteria.

Data extraction was performed systematically from all included studies, focusing on critical aspects such as study design, population characteristics, intervention details (e.g., target temperature and cooling methods), control groups, and key outcomes (mortality and neurological function). Two independent reviewers extracted key outcomes, including survival rates, neurological outcomes at various follow-up periods, adverse effects, and methodological details (such as blinding and randomization). Discrepancies were resolved by consensus discussion or a third reviewer. The extracted data were synthesized using a narrative approach due to the heterogeneity in study designs, cooling protocols, and outcome measures. For studies with comparable designs and outcome measures, we highlighted trends in results, focusing on the evolution of evidence from early studies in the 2000s to more recent trials. The synthesis aimed to contrast findings between studies using different target temperatures (e.g., 33°C vs. 36°C) and examine the differences in patient outcomes based on these temperature ranges.

A risk of bias assessment was conducted for each study. Many studies showed moderate-to-high selection and performance bias due to small sample sizes, unclear randomization, and lack of proper blinding. Detection bias occurred in studies without blinded outcome assessments, while attrition bias was generally low as most studies accounted for follow-up. Some reporting bias was present, and not all outcomes were fully reported. These biases indicate that while some studies supported hypothermia, their findings may be unreliable due to methodological limitations. These studies are summarized in Table 2.

**Table 2:** Risk of bias assessment for all studies included in this review

Study	Selection bias	Performance bias	Detection bias	Attrition bias	Reporting bias
Bernard et al. [19]	Moderate: small sample size, possible bias in randomization	High: limited blinding, high risk of performance bias	High: outcome assessments not clearly blinded	Moderate: incomplete reporting on dropout rates	High: potential selective reporting of outcomes
Hypothermia After Cardiac Arrest Study Group [20]	Low: adequately described random sequence generation and allocation	Moderate: blinding not explicitly described	Low: likely blinded outcome assessments	Low: accounted for in the analysis, minimal bias	Low: no evidence of selective reporting
Hachimi-Idrissi et al. [21]	Moderate: small study, not enough details on randomization	Moderate: blinding not clear, potential bias	Moderate: blinding not described, detection bias possible	Moderate: some patients lost to follow-up	Moderate: reporting bias possible; not all outcomes are clear
Laurent et al. [22]	Moderate: the randomization process is not well-described	High: no mention of blinding, high-performance bias	High: detection bias likely, not clearly controlled	Moderate: high dropout rates, not well-addressed	High: selective reporting likely, no full data
Bernard et al. [23]	Low: proper randomization, but sample size limits power	Moderate: blinding process not clearly described	Moderate: detection bias unclear	Low: minimal attrition, well-documented	Low: no selective reporting noted
Nielsen et al. [24]	Low: strong randomization and allocation concealment	Low: proper blinding of healthcare providers and assessors	Low: outcome assessors properly blinded	Low: minimal loss to follow-up, transparent reporting	Low: all outcomes reported transparently
Kim et al. [25]	Low: randomization well-explained, no significant bias detected	Moderate: blinding unclear, risk of performance bias	Moderate: detection bias risk due to unclear blinding	Low: minimal attrition with full reporting	Low: no selective reporting detected
Kirkegaard et al. [26]	Low: adequately randomized and allocated, low risk of bias	Low: well-blinded, reducing performance bias	Low: outcome assessors are blinded, minimizing detection bias	Low: transparent reporting and low attrition	Low: full reporting of planned outcomes
Lascarrou et al. [27]	Low: well-controlled study design, low risk of selection bias	Low: blinding well-implemented, minimizing bias	Low: adequately blinded outcome assessment	Low: minimal attrition documented	Low: transparent and complete reporting
Dankiewicz et al. [28]	Low: extensive sample size and strong randomization	Low: blinding maintained throughout the trial	Low: outcome assessment well-blinded	Low: low attrition rates and clear reporting	Low: no reporting bias observed
Le May et al. [29]	Low: proper randomization with no significant bias	Low: proper blinding maintained	Low: proper outcome assessor blinding	Low: minimal dropout, well-accounted for	Low: all outcomes reported without bias
Kim et al. [30]	Moderate: an observational study, potential for selection bias	High: observational study, no blinding	High: no blinding of outcome assessment	Moderate: higher attrition due to the observational nature	Moderate: selective reporting is possible due to the design
Kim et al. [31]	Moderate: observational design, inherent selection bias possible	High: observational, no blinding in practice	High: outcome assessment not blinded	Moderate: potential for attrition bias in observational setup	Moderate: reporting bias possible in observational setup
Lee et al. [32]	Moderate: retrospective study, not randomized, selection bias present	High: retrospective, no blinding possible	High: no blinding possible in retrospective analysis	Moderate: incomplete follow-up due to retrospective design	High: selective reporting likely due to retrospective design
Slagle et al. [33]	Moderate: retrospective design with moderate selection bias	Moderate: blinding is not possible due to the retrospective design	Moderate: detection bias is likely given by the study design	Moderate: attrition rates higher, not fully addressed	Moderate: some selective reporting is likely in this design
Tanaka et al. [34]	Low: large cohort, well-controlled, low risk of selection bias	Low: blinding adequate given study design	Low: proper detection bias controls implemented	Low: well-documented attrition and follow-up	Low: no selective reporting observed

Results

Early Studies (2000-2010)

Extensive studies about the benefits of induced mild hypothermia were conducted on animal models in the 1990s [18]. These studies found that hypothermia offered neuroprotection following cerebral ischemia, cardiac arrest, and bacterial meningitis in animals. In humans, a study found that hypothermia provides some benefits in patients with severe brain injury to control traumatic intracranial hypertension. As a result, induced hypothermia was added as a treatment modality for patients with brain injury. Following this, two prospective randomized controlled trials were conducted to evaluate the benefit of induced hypothermia in patients experiencing OHCA.

The first study was published by Bernard et al. in 2002 [19]. The study included 77 patients randomized to either normothermia or a reduction in core body temperature to 33°C. Basic cooling measures initiated hypothermia in the ambulance, followed by active surface cooling after reaching the hospital. The study measured satisfactory neurologic function at discharge. It found that 21 of the 43 patients treated with hypothermia survived and had a good neurologic outcome (49%) compared with only nine of 34 treated with normothermia (26%) (p = 0.046). After adjustment, the calculated odds ratio for a satisfactory outcome with hypothermia compared to normothermia was 5.25 (95% confidence interval, CI, 1.47-18.76; p = 0.011). Additionally, the mortality rate in the hypothermia group was 51%, while in the normothermia group, it was 68%. The authors also concluded that there were no clinically significant adverse effects due to the induced hypothermia for 12 hours.

Another study by the Hypothermia After Cardiac Arrest (HACA) group was published in 2002 [20]. This study evaluated 275 patients who suffered an OHCA due to ventricular fibrillation (VF). Patients were randomly assigned to the hypothermia group with a target temperature of 32°C to 34°C or the normothermia group. The study found that 75 of 136 patients (55%) had a favorable neurologic outcome in the hypothermia group compared with 54 of 137 patients (39%) in the normothermia group, with a risk ratio of 1.40 (95% CI, 1.08-1.81). The hypothermia group was also found to have lower mortality after six months, 41% compared to 55%, with a risk ratio of 0.74 (95% CI, 0.58-0.95). Finally, there was no significant difference in the rate of adverse effects. These studies conducted in the early 2000s showed a significant difference in neurological outcomes and survivability when hypothermia was induced at 32°C to 34°C [19,20]. These early studies formed much of the basis of guidelines for the induction of hypothermia in cardiac arrest patients to improve neurological outcomes and survivability [35]. These studies are summarized in Table 3.

**Table 3:** Summary of key studies from 2002 to 2005 on hypothermia for OHCA patients RCT: randomized controlled trial; OHCA: out-of-hospital cardiac arrest

Study	Study design	Year	Location	Number of patients	Inclusion criteria	Summary
Bernard et al. [19]	RCT	2002	Australia	77	OHCA due to ventricular fibrillation	Significant reduction in mortality and significantly better neurological outcome in the hypothermia group
Hypothermia After Cardiac Arrest Study Group [20]	RCT	2002	Europe	275	OHCA due to any shockable rhythm	Mortality was lower in the hypothermia group, along with a favorable neurological outcome
Hachimi-Idrissi et al. [21]	RCT	2005	Europe	61	OHCA due to any cause	Lower mortality and better neurological outcomes were seen in the hypothermic group
Laurent et al. [22]	RCT	2005	Europe	61	OHCA due to any cause	Higher mortality rates and more adverse events were observed in hypothermic patients compared to normothermia patients

In 2005, Hachimi-Idrissi et al. conducted a small clinical study on 61 patients, all treated according to the ERC guidelines for basic and advanced life support [21]. The patients were randomized into two groups: hypothermic (33°C) and normothermic (37°C). Hypothermic patients showed improved neurological outcomes and a higher survival rate than normothermic patients [21]. The authors suggested that hypothermia might interrupt pathways involved in anoxic brain injury, which could explain the better neurological outcomes observed in these patients.

Another study was conducted in late 2005 by Laurent et al. to observe the survival outcomes of two groups (61 patients) over six months: one normothermic and one hypothermic [22]. A temperature of 32°C was maintained in one group and 36°C in the other. Interestingly, the six-month survival rates were 32% and 45% for the normothermic and hypothermic groups, respectively. However, the neurological outcomes were favorable in both groups. Hypokalemia was reported in an equal number of patients from both groups, while hypophosphatemia was reported more frequently among the hypothermic group. The authors also noted that during the first 24 hours in the ICU, six hypothermic patients experienced ventricular tachycardia compared to two normothermic patients [22].

Recent Trials (2010-2024)

The 2002 trials were followed by the Rapid Infusion of Cold Hartmann's trial by Bernard et al. in 2010 [23]. This trial involved patients who suffered cardiac arrest due to VF and were resuscitated out of the hospital. A total of 234 patients were randomized to either receive paramedic cooling or hospital cooling measures. The paramedic cooling group received a rapid infusion of 2 L of ice-cold lactated Ringer's solution, resulting in a mean decrease of 0.8°C in core temperature. The study found no significant difference in favorable outcomes at hospital discharge between the paramedic-cooled and hospital-cooled groups, with a risk ratio of 0.90 (95% CI, 0.70-1.17).

The TTM trial, conducted by Nielsen et al. in 2013 [24], compared the effects of maintaining a body temperature at 33°C and 36°C after OHCA. This major trial included 950 patients from 36 intensive care units in Europe and Australia. The patients were randomly assigned to a target temperature of either 33°C or 36°C. At the end of the trial, 235 out of 473 patients (50%) in the 33°C group had died compared to 255 out of 466 (48%) in the 36°C group (hazard ratio, 1.06; 95% CI, 0.89-1.28). The study also measured mortality and neurological function at a six-month follow-up. In the 33°C group, 54% of the patients had died or had poor neurological function compared with 52% in the 36°C group. It was also concluded that hypokalemia was significantly more common in the hypothermia group.

In 2014, Kim et al. conducted a randomized controlled trial involving 1,359 patients to assess prehospital cooling [25]. The prehospital cooling group received 2 L of 4°C normal saline, while the other group received standard care. Standard care involves the usual medical management provided to patients in a prehospital setting without the application of specific cooling interventions [25]. The mean reduction in temperature due to the intervention was 1.20°C in VF patients and 1.30°C in non-VF patients. Of the VF patients who received prehospital cooling, 62.7% (95% CI, 57.0-68.0) survived compared to 64.3% (95% CI, 58.6-69.5) of patients who did not receive prehospital cooling (p = 0.69). Similar results were seen in the non-VF subgroup, where 19.2% of the intervention group survived compared to 16.3% of the standard care group (p = 0.30). There was no significant difference in neurological outcomes between the intervention and control groups. The only statistically significant outcome was that the intervention group experienced rearrests in the field more commonly than the control group (26% vs. 21%, p = 0.008).

Kirkegaard et al. conducted a study in 2017 to determine the optimal duration of targeted hypothermia [26]. This randomized controlled trial was conducted in 10 ICUs across six European countries. They randomized 335 patients to 24 or 48 hours of a target temperature of 33°C. At the six-month follow-up, 120 out of 175 patients (69%) in the 48-hour group had favorable neurological outcomes compared to 112 out of 176 (64%) in the 24-hour group, with a relative risk of 1.08 (95% CI, 0.93-1.25; p = 0.33). No significant difference was found in mortality between the groups; however, adverse effects were significantly more common in the 48-hour group (97% vs. 91%; relative risk, 1.06; 95% CI, 1.01-1.12; p = 0.04). The risk of hypotension was higher in the 48-hour group, while the risk of bleeding was higher in the 24-hour group.

In 2019, a randomized controlled trial was performed on 581 patients to evaluate outcomes at the end of three months [27]. On day 90, 29 out of 284 hypothermia patients (10.2%) were alive compared to 17 out of 297 normothermic patients (5.7%) (95% CI, 0.1-8.9; p = 0.04). It was also observed that hypothermic patients had more favorable neurological outcomes at day 90 compared to normothermic patients. These studies are summarized in Table 4.

**Table 4:** Summary of key studies from 2010 to 2019 on hypothermia for OHCA patients RCT: randomized controlled trial; OHCA: out-of-hospital cardiac arrest; VF: ventricular fibrillation

Author	Study design	Year	Location	Number of patients	Inclusion criteria	Summary
Bernard et al. [23]	RCT	2010	Australia	234	OHCA due to VF	No significant difference was found between the paramedic and hospital-cooled groups
Nielsen et al. [24]	RCT	2013	Australia, Europe	950	OHCA with a cardiac cause	No significant difference in mortality and neurologic outcome between 33°C and 36°C
Kim et al. [25]	RCT	2014	United States	1,359	OHCA due to any rhythm	No significant difference in mortality or neurologic outcomes in patients receiving prehospital cooling compared to standard of care
Kirkegaard et al. [26]	RCT	2017	Europe	355	OHCA due to any rhythm	No significant difference in mortality and neurological outcomes between hypothermia for 24- or 48-hour subgroups
Lascarrou et al. [27]	RCT	2019	Europe	581	OHCA survivors	Hypothermic patients had a higher survival rate as well as better neurological outcomes compared to normothermic patients

Trials published from 2021 onward show a clear trend of no significant difference in outcomes between targeted hypothermia and targeted normothermia. The first of these studies, published by Dankiewicz et al. in 2021 as a follow-up to the TTM trial (subsequently called the TTM2 trial), randomized 1,900 patients to undergo targeted hypothermia at 33°C followed by controlled rewarming or targeted normothermia with early treatment of fever [28]. At the six-month follow-up, mortality in the hypothermia group was 465 out of 925 (50%) compared to 446 out of 925 (48%) in the normothermia group, with a relative risk of hypothermia of 1.04 (95% CI, 0.94-1.14; p = 0.37). Similarly, 488 out of 881 patients (55%) had a moderately severe disability in the hypothermia group compared to 479 out of 866 (55%) in the normothermia group, with a relative risk of hypothermia of 1.00 (95% CI, 0.92-1.09). Another significant finding of the study was that arrhythmias resulting in hemodynamic compromise were more common in the hypothermia group (24% vs. 17%; p < 0.001). This was the most extensive study comparing targeted hypothermia vs. normothermia and concluded that there is no significant difference in clinical outcomes for targeted hypothermia.

Another study by Le May et al., the Moderate vs. Mild Therapeutic Hypothermia in Comatose Survivors of Out-of-Hospital Cardiac Arrest trial, was released shortly afterward [29]. The study compared mortality and neurologic outcomes 180 days after cardiac arrest in patients undergoing targeted moderate hypothermia (31°C) and mild hypothermia (34°C). Statistically speaking, the results were nonsignificant as the mortality rate and poor neurological outcomes were 48.4% and 45.4% in the 31°C and 34°C groups, respectively.

In the following year, 2022, three separate trials were completed in South Korea. The first, published by Kim et al., included 1,339 patients, of which 1,054 were treated at 33°C, while 285 were treated at 36°C [30]. No statistically significant difference was found in neurological outcomes after six months. The adjusted odds ratio was 0.97 (95% CI, 0.73-1.29). The difference in survival after six months was also insignificant, with an adjusted hazard ratio of 1.08 (95% CI, 0.91-1.28).

In a second study of 1,566 patients by Kim et al., there was no significant difference in neurological outcomes or survival to hospital discharge [31]. In the TTM group, 1.8% of the patients had a good neurological outcome compared to 2.9% in the non-TTM group (p = 0.183). Similarly, survival to hospital discharge was 2.9% in the TTM group and 3.4% in the non-TTM group (p = 0.666).

The third study by Lee et al. applied TTM with a temperature feedback system to 318 patients, while there were 87 patients in the normothermia group [32]. No statistically significant differences were seen after one month in neurological outcomes (odds ratio, 0.99; 95% CI, 0.56-1.25) and survival rate (odds ratio, 1.25; 95% CI, 0.99-1.78).

Over time, many have speculated that preventing fever is the critical intervention in OHCA, suggesting that true hypothermia might not be necessary [33]. A study by Slagle et al. analyzed 634 patients between 2010 and 2019 to evaluate neurologic outcomes [33]. Hypothermia was induced in one group of 473 patients at 33°C, while the other 161 patients received targeted normothermia at 36.5°C. Patients in the hypothermia group had higher odds of more favorable neurologic outcomes than those in the normothermia group (odds ratio, 2.4; 95% CI, 1.3-4.6; p = 0.006). However, there was no statistically significant difference between the two groups regarding mortality or ICU/ventilator days. Interestingly, patients in the normothermia group had a longer length of stay than those in the hypothermia group.

Recently, a seven-year-long multicenter cohort study conducted in Japan reported their findings after evaluating the effects of hypothermia among OHCA survivors [34]. Upon analysis of 2,936 patients, the survival rate was significantly higher for hypothermia patients (p < 0.01) compared to normothermia patients. The hypothermic patients also had more favorable neurological outcomes than the other group. These studies are summarized in Table 5.

**Table 5:** Summary of key studies from 2021 to 2024 on hypothermia for OHCA patients RCT: randomized controlled trial; OHCA: out-of-hospital cardiac arrest

Study	Study design	Year	Location	Number of patients	Inclusion criteria	Summary
Dankiewicz et al. [28]	RCT	2021	Multiple	1,850	OHCA due to any cause	No significant difference in mortality and neurological outcomes at a six-month follow-up between a targeted management of 33°C or normothermia
Le May et al. [29]	RCT	2021	Canada	367	OHCA survivors	No statistically significant difference in mortality or neurological outcomes after six months when comparing targeted management at 31°C vs. 34°C
Kim et al. [30]	Observational	2022	South Korea	1,339	OHCA survivors	No statistically significant difference in survival and neurological outcomes
Kim et al. [31]	Observational	2022	South Korea	1,566 (783 matched pairs)	OHCA survivors	No statistically significant difference in survival and neurological outcomes
Lee et al. [32]	Retrospective analysis	2022	South Korea	405	OHCA survivors	No statistically significant difference in survival and neurological outcomes after one month
Slagle et al. [33]	Retrospective analysis	2023	United States	634	OHCA survivors	Better neurological outcomes were seen in hypothermia patients, but no significant difference in mortality between the two groups
Tanaka et al. [34]	Multicenter cohort study	2024	Japan	2,936	OHCA survivors	The survival rate was much higher in hypothermia-treated patients compared to normothermia patients

Discussion

Future of TTM and Recommendations for Healthcare Providers

As evident from the discussion above, the latest evidence for the benefits of induced hypothermia is largely inconclusive. TTM with a goal of hypothermia was recommended in the early 21st century, based on early studies by Bernard et al. and the HACA study group [19,20]. However, the results of recent randomized controlled trials have pointed in another direction. Many authors have highlighted various shortcomings of the earlier trials, including small sample sizes, high risk of bias due to unblinded physicians, lack of standardized protocols, and the mean temperature in the normothermia group being 37.5°C. The high temperature in the normothermia group raises concerns about neurological injury due to hyperthermia independent of cardiac arrest [36].

The TTM and TTM2 trials were major follow-up trials with standardized protocols. Both trials were extensive international studies with 950 and 1,850 subjects, respectively. Along with multiple other randomized controlled trials, the conclusion reached by both was that there is no significant difference in neurological outcomes and mortality between targeted hypothermia and targeted normothermia. On the contrary, multiple authors have concluded that adverse effects are more common in the hypothermia group, including higher rates of rearrest, hypokalemia, arrhythmias leading to hemodynamic compromise, and hypotension [24-27].

The TTM2 trial, conducted by Dankiewicz et al. [28], demonstrated that while no significant difference was observed in terms of improved neurological outcomes or survival rates between hypothermic and normothermic groups, adverse effects such as arrhythmias resulting in hemodynamic compromise were more common in the hypothermia group (24% vs. 17%). Additionally, studies by Kim et al. [25] and Kirkegaard et al. [26] reported increased incidences of hypokalemia, hypotension, bleeding, and even rearrest during the cooling process. These adverse effects highlight the potential risks of therapeutic hypothermia, which may outweigh the perceived benefits in some cases. As such, the future of targeted hypothermia must be reevaluated, as the supporting evidence for this recommendation is weak. The blanket guidelines for inducing hypothermia in cardiac arrest survivors have the potential to cause unnecessary adverse effects and represent poor resource management for healthcare systems. While standardized guidelines are important, recent evidence suggests an individualized approach to post-resuscitative temperature management may help optimize outcomes and minimize unnecessary adverse effects rather than relying solely on blanket hypothermia protocols. The results of this review align with other systematic reviews and meta-analyses published in the last two years by Granfeldt et al. [6], Bisht et al. [8], Aneman et al. [37], and Fernando et al. [38]. This trend is concerning as more evidence contradicts current guidelines. More research must be conducted to conclusively prove these guidelines or gather enough evidence to modify them.

While earlier studies on therapeutic hypothermia provided the foundation for its inclusion in the early guidelines, the latest evidence highlights various biases and limitations. Future randomized controlled trials should include larger sample sizes to enhance statistical power. They should also ensure greater population diversity (age, sex, race, and preexisting conditions), as this will provide a more comprehensive understanding of how TTM may affect different subgroups. Earlier studies often lacked this diversity, which may limit the generalizability of their findings. Second, one of the primary biases identified in early studies was the elevated mean temperature (often around 37.5°C) in the normothermia control group. Future studies should ensure stricter control over normothermia, targeting a temperature range below the threshold for hyperthermia (e.g., 36°C or below). This will allow researchers to assess the true effect of hypothermia compared to well-controlled normothermia without the confounding factor of hyperthermia. Third, the lack of standardized cooling protocols in earlier studies led to discrepancies in the results. Future studies should establish uniform criteria for the initiation, duration, and cooling method (surface cooling vs. intravascular cooling) to allow for better comparison between studies. This includes defining clear start times (e.g., upon return of spontaneous circulation) and cooling durations, such as comparing 24 hours vs. 48 hours of cooling to determine the optimal strategy. Many previous trials measured outcomes only at short-term follow-ups (e.g., hospital discharge or six months). Given that neurological damage and recovery may take longer to manifest, future studies should include longer follow-up periods, ideally over 12 months or more, to assess long-term neurological outcomes and quality of life in OHCA survivors. Additionally, advanced imaging and biomarker analysis can offer insights into the neuroprotective mechanisms of TTM, and studies should explore whether avoiding hyperthermia is more beneficial than inducing hypothermia. Collectively, these improvements in study design will help provide more specific and reliable recommendations for clinicians managing temperature control in OHCA survivors.

Limitations

The limitations of this review article include the inclusion of OHCA survivors regardless of the cause, which may introduce variability in the efficacy of induced hypothermia due to different root causes. Additionally, TTM has a broad definition, and induced hypothermia could be compromised due to factors localized to individual hospital settings. Finally, IHCA survivors were not included in the review, as they have immediate access to care, and their mortality and neurological outcomes can differ significantly.

## Conclusions

Managing cardiac arrest survivors is a complex task requiring precise guidelines to minimize neurological damage and improve mortality. Although there are evidence-based guidelines for immediate steps after a cardiac arrest, the guidelines are not as clear on the best course of action for post-resuscitative care. We explored one aspect of OHCA survivors, targeted hypothermia, and found no significant difference in mortality and neurological outcomes in nine out of 16 studies. Two studies that showed beneficial outcomes were published in the early 2000s and had a high risk of bias, as pointed out by multiple authors.

Based on the latest evidence, including large-scale randomized controlled trials such as TTM2, there is no definitive benefit of hypothermia over normothermia in improving neurological outcomes or survival in OHCA patients. Current findings suggest that TTM should focus on avoiding hyperthermia rather than inducing hypothermia. Therefore, we recommend a more individualized approach to temperature management, tailoring interventions to the specific needs of each patient rather than applying a standardized hypothermia protocol.

This review aims to spark a discussion on existing guidelines as the evidence for them continues to weaken with each subsequent randomized controlled trial. The studies from the early 2000s by Bernard et al. and the HACA study group need to be revisited and reevaluated to identify discrepancies that might have affected their results. Finally, more research must be conducted to determine the most effective treatment modality: targeted hypothermia, normothermia, or simply avoiding hyperthermia.
